# Japanese encephalitis virus infection induces changes of mRNA profile of mouse spleen and brain

**DOI:** 10.1186/1743-422X-8-80

**Published:** 2011-02-24

**Authors:** Yang Yang, Jing Ye, Xiaohong Yang, Rong Jiang, Huanchun Chen, Shengbo Cao

**Affiliations:** 1 State Key Laboratory of Agricultural Microbiology, Huazhong Agricultural University, Wuhan, Hubei 430070, PR China; 2 Laboratory of Animal Virology, College of Veterinary Medicine, Huazhong Agricultural University, Wuhan, Hubei 430070, PR China

## Abstract

**Background:**

Japanese encephalitis virus (JEV) is a mosquito-borne flavivirus, leading to an acute encephalitis and damage to the central nervous system (CNS). The mechanism of JEV pathogenesis is still unclear. DNA microarray analyses have been recently employed to detect changes in host gene expression, which is helpful to reveal molecular pathways that govern viral pathogenesis. In order to globally identify candidate host genes associated with JEV pathogenesis, a systematic mRNA profiling was performed in spleens and brains of JEV-infected mice.

**Results:**

The results of microarray analysis showed that 437 genes in spleen and 1119 genes in brain were differentially expressed in response to JEV infection, with obviously upregulated genes like pro-inflammatory chemokines and cytokines, apoptosis-related proteases and IFN inducible transcription factors. And the significant pathways of differentially expressed genes are involved in cytokine-cytokine receptor interaction, natural killer cell mediated cytotoxicity, antigen processing and presentation, MAPK signaling, and toll-like receptor signaling, etc. The differential expression of these genes suggests a strong antiviral response of host but may also contribute to the pathogenesis of JEV resulting in encephalitis. Quantitative RT-PCR (RT-qPCR) assay of some selected genes further confirmed the results of microarray assay.

**Conclusions:**

Data obtained from mRNA microarray suggests that JEV infection causes significant changes of mRNA expression profiles in mouse spleen and brain. Most of differentially expression genes are associated with antiviral response of host, which may provide important information for investigation of JEV pathogenesis and therapeutic method.

## Background

Japanese encephalitis virus (JEV), a mosquito-borne flavivirus belonging to family *Flaviviridae*, is responsible for an acute encephalitis and damage to the central nervous system (CNS) in wide areas of southern and eastern Asia. And recently, it has been isolated from previously non-affected areas, such as Australia [1]. Japanese encephalitis (JE) has a high fatality rate of 30% and around half of the JE survivors have severe neurological sequelae [2]. Approximately 50,000 JE cases with 10,000 deaths are reported annually [3]. Following entry into the host system through a mosquito bite, JEV may replicates in various organs such as liver and spleen, and then reaches the central nervous system, resulting in a rapid inflammatory response [4]. According to the observations from studies of other flaviviruses, specifically dengue virus, it has been proposed that JEV traverse through a lymphatic route that also involves cells of the monocyte/macrophage lineage. Recently, JEV has been shown to effectively replicate within lymphocytes and macrophages, thereby making these cell types possible carriers of the virus from the periphery to the CNS [5,6]. However, it remains to be elucidated how JEV infects the CNS via these peripheral cells. In addition, although neurological disorders caused by JEV are often characterized by evidence of immune system recognition and the presence of inflammatory components among the neuropathological changes, the mechanisms by which this virus causes neurological disease are not fully understood [7].

Recently, multiple DNA microarray analyses have been employed to detect changes in host gene expression after viral infection, which makes it possible to reveal molecular pathways that govern viral pathogenesis. Genechip analysis of human umbilical vein endothelial cells infected with Dengue Virus (DV) detected the upregulation of 269 genes and downregulation of 126 genes [8]. Gene profiling study of West Nile Virus (WNV) infected human embryonic kidney cells, human glioma cells and mice tissues were also performed [9,10]. Furthermore, identification of gene profiles in JEV-infected neuroblastoma cells and brain tissue have been reported recently, suggesting an increased expression of pro-inflammatory cytokines, chemokines, and anti-viral response genes after JEV infection [11,12]. However, both of studies on JEV were restricted to CNS, and few gene profiling researches about response in peripheral immune system has been carried out.

In present study, to globally identify candidate host genes associated with JEV pathogenesis, DNA microarray technology was utilized to investigate mRNA profile in spleen and brain tissues of mice infected with JEV wild strain P3, and some of the selected genes were further confirmed by quantitative RT-PCR. It was demonstrated that JEV infection resulted in significant changes in the expression of numerous genes in spleen and brain tissues, which could be crucial messages for revealing of JEV pathogenesis.

## Results

### mRNA expression profile of JEV-infected mice

**Figure 1:**
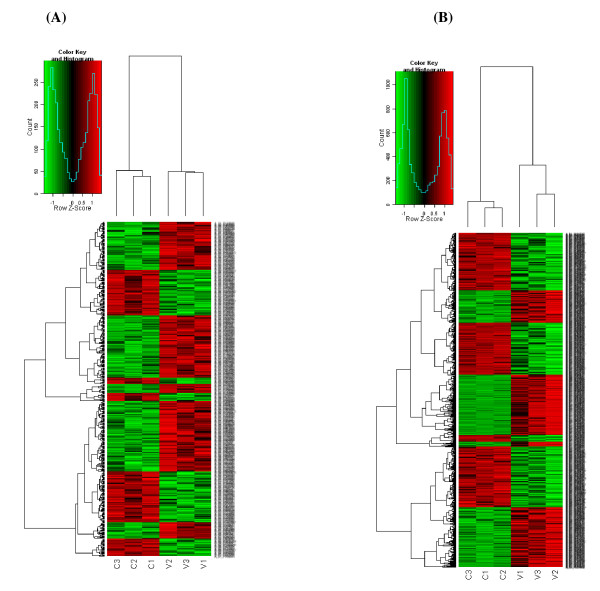
**Unsupervised hierarchical clustering of differentially expressed mRNAs**. mRNA hybridization was performed with the use of 4 × 44 K Agilent Whole Mouse Genome Oligo Microarray. For each sample pair, the experiments were done with two independent hybridizations (Cy3 and Cy5 interchanging labeling). Genes that had ≥ |2|-fold change were identified as significantly differentially expressed. Differentially regulated genes were clustered using SAS (ShanghaiBio Analysis System) to identify significant gene expression patterns in spleens (A) and brains (B) of JEV-infected mice. Red indicates higher expression and green indicates lower expression in JEV-infected mice versus control. Black indicates no expression difference. The small figure represents color scales used in the cluster map.C indicates control group and V indicates viral-inoculated group. Each group contains 3 mice.

**Figure 2:**
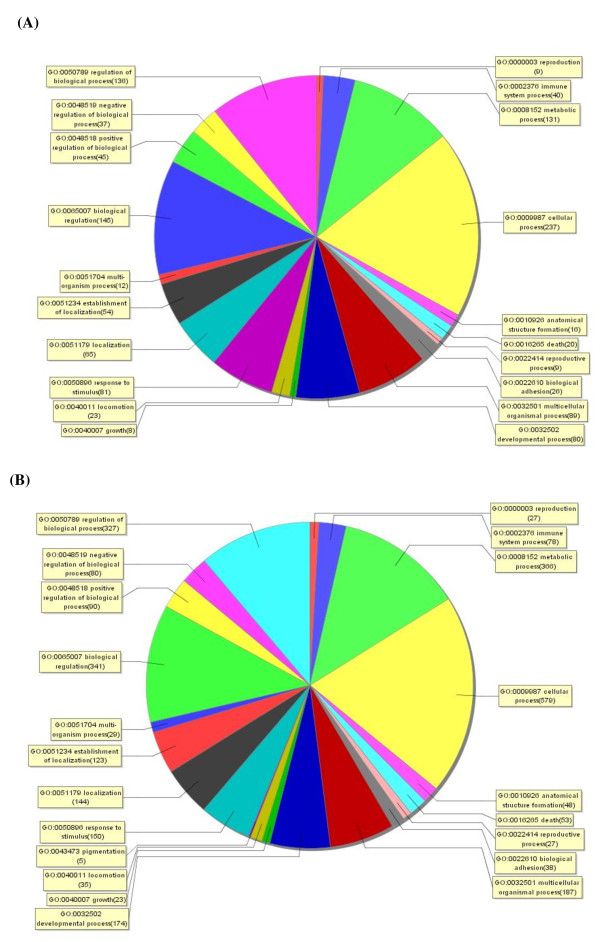
**Enriched Gene Ontology terms in the biological process category among differentially expressed genes**. After mRNA microarray assay, significantly enriched Gene Ontology analysis in the biological process category among differentially expressed genes (fold change ≥ 2.0) in spleens (A) and brains (B) of JEV-infected mice was performed by using SAS (ShanghaiBio Analysis System). Each color section represents a different biological process and the gene number enriched in this section was shown following the biological process name.

A mouse whole gene array was used to perform a systematic analysis of mRNA expression profile of spleen and brain tissues of JEV P3-infected mice which were sacrificed at day 3 and day 6 post-inoculation respectively. Genes that had ≥ |2|-fold change were identified as significantly differential expression. Of 41174 genes represented on the chip, 437 genes were differentially expressed in mouse spleens and 1119 genes were differentially regulated in brains in response to JEV infection (change fold ≥ 2.0, *p *value < 0.05). Unsupervised clustering (Figure 1) analysis of the expression profiles showed a distinct mRNA signature in both spleens and brains during JEV infection. To elucidate the correlation between gene expression pattern and JEV infection-induced biological processes, functional classification of mRNA transcripts and pathway analysis were performed. Differentially regulated genes in spleens of JEV-infected mice are involved in the biological processes such as cellular process, biological regulation and immune system process, etc (Figure 2A). And the significant pathways of differentially expressed genes are known to be involved in cytokine-cytokine receptor interaction, natural killer cell mediated cytotoxicity, antigen processing and presentation, and chemokine signaling, etc (Table 1). While biological processes which showed differentially regulated genes in brains of JEV infected mice are cellular process, metabolic process, and biological regulation, etc (Figure 2B). And the significant pathways of differentially expressed genes in mouse brain are cytokine-cytokine receptor interaction, MAPK signaling, neuroactive ligand-receptor interaction, and toll-like receptor signaling, etc. (Table 2).

**Table 1:** Significant pathways of the differential expressed genes in spleens of JEV-infected mice.

Pathway name	No. of genes	*p*-Value
Antigen processing and presentation	9	0
Chemokine signaling pathway	13	0
Complement and coagulation cascades	7	0
Cytokine-cytokine receptor interaction	23	0
Natural killer cell mediated cytotoxicity	9	0
Classical Complement Pathway	3	1.00E-04
Complement Pathway	3	4.00E-04
Alternative Complement Pathway	2	0.0021
Granzyme A mediated Apoptosis Pathway	2	0.0036
Toll-like receptor signaling pathway	4	0.0071
Intestinal immune network for IgA production	3	0.009
CCR3 signaling in Eosinophils	2	0.0111
Caspase Cascade in Apoptosis	2	0.0121
NOD-like receptor signaling pathway	3	0.0121
MAPK signaling pathway	6	0.0168
ECM-receptor interaction	3	0.0232
Jak-STAT signaling pathway	4	0.0284
IFN gamma signaling pathway	1	0.0478
B Lymphocyte Cell Surface Molecules	1	0.0611
T cell receptor signaling pathway	3	0.069

**Table 2:** Significant pathways of the differential expressed genes in brains of JEV-infected mice.

Pathway name	No. of genes	*p*-value
Chemokine signaling pathway	19	0
Cytokine-cytokine receptor interaction	30	0
Jak-STAT signaling pathway	19	0
MAPK signaling pathway	19	0
NOD-like receptor signaling pathway	10	0
Purine metabolism	14	0
Toll-like receptor signaling pathway	13	0
IL-2 Receptor Beta Chain in T cell Activation	5	6.00E-04
Neuroactive ligand-receptor interaction	16	6.00E-04
NF-kB activation by Nontypeable Hemophilus influenzae	4	0.0012
Apoptosis	7	0.0023
Antigen processing and presentation	7	0.0037
IFN gamma signaling pathway	2	0.0082
Natural killer cell mediated cytotoxicity	8	0.011
T cell receptor signaling pathway	7	0.0123
IFN alpha signaling pathway	2	0.0156
FAS signaling pathway (CD95)	3	0.0173

### Genes with differential expression in spleens of JEV-infected mice

In spleens of mice with JEV infection, 263 genes were detected to be significantly upregulated and 174 genes were downregulated (Table 3). Genes with increased expression in spleens of JEV infected mice mainly fell into the function of immune response to viral infections. These include pro-inflammatory cytokine IFN-γ, IFN response transcription factor IRF7, IFN-induced proteins like IFIT1, IFITM3 and IFITM7, protein degradation gene ubiquitin-specific protease Usp29 and Usp18, apoptosis related genes granzymA, granzymeB, Porferin, and IMNB2, killer cell lectin-like receptors KLRC1, KLRC2 and KLRC3, and chemokines such as CXCL10, CXCL11, CCL12, CCL2 and CCL9. The marked increase in expression of these genes implies the occurrence of a strong antiviral protective response to JEV infection. The significantly downregulated genes are mainly involved in cell adhesion molecules such as monocyte/macrophage-lineage cell marker CD163, transmembrane cell surface receptor of Langerhans cells CD207, and ligand for myeloid cells receptor CD200. Evidence was also found for decreased expression of interferon transcription factor IRF6 and interleukin 7 receptor, which may contribute to JEV pathogenesis as well.

**Table 3:** Differentially-regulated genes of our interest in spleens of mice with JEV Infection

Genbank Accession	Gene symbol	Gene description	Fold change	*p*-Value
**Up-regulated**				
NM_177261	Kndc1	kinase non-catalytic C-lobe domain (KIND) containing 1	21.440	0.0252
NM_019494	Cxcl11	chemokine (C-X-C motif) ligand 11	19.752	0.0084
NM_013542	Gzmb	granzyme B	6.202	0.0286
NM_031167	Il1rn	interleukin 1 receptor antagonist (Il1rn), transcript variant 1	6.060	0.0015
AK157531	LOC629091	activated spleen cDNA, RIKEN full-length enriched library, clone:F830221E13	5.500	0.0154
NM_001033228	Itga1	integrin alpha 1	5.482	0.0308
NM_013584	Lifr	leukemia inhibitory factor receptor	5.319	0.0109
NM_177923	H2-M10.2	histocompatibility 2, M region locus 10.2	5.276	0.0009
NM_008198	Cfb	complement factor B	5.188	0.0019
NM_001005858	LOC667370	similar to interferon-induced protein with tetratricopeptide repeats 3	4.949	0.0459
NM_145153	Oas1f	2'-5' oligoadenylate synthetase 1F	4.619	0.0013
NM_011331	Ccl12	chemokine (C-C motif) ligand 12	4.539	0.0308
NM_027893	Pvrl4	poliovirus receptor-related 4	4.429	0.0352
NM_010741	Ly6c	lymphocyte antigen 6 complex, locus C	4.287	0.0042
NM_010370	Gzma	granzyme A	4.236	0.0010
NM_009152	Sema3a	sema domain, immunoglobulin domain (Ig), short basic domain, secreted, (semaphorin) 3A	4.132	0.0358
AF229257	Usp29	ubiquitin-specific processing protease 29	4.033	0.0093
NM_133871	Ifi44	interferon-induced protein 44	3.843	0.0053
NM_009912	Ccr1	chemokine (C-C motif) receptor 1	3.734	0.0013
BC025535	Fcgr1	Fc receptor, IgG, high affinity I	3.668	0.0096
NM_145226	Oas3	2'-5' oligoadenylate synthetase 3	3.531	0.0017
NM_008331	Ifit1	interferon-induced protein with tetratricopeptide repeats 1	3.433	0.0460
NM_021274	Cxcl10	chemokine (C-X-C motif) ligand 10	3.418	0.0307
NM_016850	Irf7	interferon regulatory factor 7	3.378	0.0083
NM_008530	Ly6f	lymphocyte antigen 6 complex, locus F	3.313	0.0027
NM_011940	Ifi202b	interferon activated gene 202B	3.296	0.0266
NM_145211	Oas1a	2'-5' oligoadenylate synthetase 1A	3.283	0.0082
NM_145227	Oas2	2'-5' oligoadenylate synthetase 2	3.178	0.0083
NM_144559	Fcgr4	Fc receptor, IgG, low affinity IV	3.069	0.0067
NM_027835	Ifih1	interferon induced with helicase C domain 1	2.940	0.0258
NM_008462	Klra2	killer cell lectin-like receptor, subfamily A, member 2	2.906	0.0001
NM_021792	Iigp1	interferon inducible GTPase 1	2.535	0.0232
L38281	Irg1	immune-responsive gene 1	2.496	0.0329
NM_008329	Ifi204	interferon activated gene 204	2.422	0.0290
NM_025378	Ifitm3	interferon induced transmembrane protein 3	2.401	0.0002
NM_021378	Klrc3	killer cell lectin-like receptor subfamily C, member 3	2.396	0.0063
NM_011909	Usp18	ubiquitin specific peptidase 18	2.333	0.0473
NM_011333	Ccl2	chemokine (C-C motif) ligand 2	2.314	0.0356
NM_028968	Ifitm7	interferon induced transmembrane protein 7	2.246	0.0014
NM_008337	Ifng	interferon gamma	2.229	0.0054
NM_011338	Ccl9	chemokine (C-C motif) ligand 9	2.155	0.0065
NM_010653	Klrc2	killer cell lectin-like receptor subfamily C, member 2	2.131	0.0076
NM_009909	Il8rb	interleukin 8 receptor, beta	2.077	0.0036
NM_010555	Il1r2	interleukin 1 receptor, type II	2.069	0.0248
NM_010652	Klrc1	killer cell lectin-like receptor subfamily C, member 1	2.025	0.0125
NM_011073	Prf1	perforin 1 (pore forming protein)	1.820	0.0326
NM_010722	Lmnb2	lamin B2	1.381	0.1976
**Down-regulated**				
NM_025989	Gp2	glycoprotein 2 (zymogen granule membrane)	0.048	0.0005
NM_053094	Cd163	CD163 antigen	0.060	0.0171
NM_177261	Kndc1	kinase non-catalytic C-lobe domain (KIND) containing 1	0.085	0.0070
NM_144943	Cd207	CD 207 antigen	0.159	0.0441
NM_010378	H2-Aa	histocompatibility 2, class II antigen A, alpha	0.184	0.0055
NM_028135	Tmem163	transmembrane protein 163	0.211	0.0196
NM_007720	Ccr8	chemokine (C-C motif) receptor 8	0.242	0.0150
NM_016851	Irf6	interferon regulatory factor 6	0.261	0.0037
NM_013509	Eno2	enolase 2, gamma neuronal (Eno2)	0.276	0.0234
NM_011780	Adam23	a disintegrin and metallopeptidase domain 23 (Adam23)	0.281	0.0009
U96752	H2-Q1	major histocompability complex Q1b	0.282	0.0247
NM_008341	Igfbp1	insulin-like growth factor binding protein 1	0.289	0.0144
NM_010565	Inhbc	inhibin beta-C (Inhbc)	0.304	0.0307
AK089361	AK089361	B6-derived CD11 +ve dendritic cells cDNA	0.307	0.0417
NM_013517	Fcer2a	Fc receptor, IgE, low affinity II, alpha polypeptide	0.315	0.0318
NM_016808	Usp2	ubiquitin specific peptidase 2 (Usp2), transcript variant 1	0.319	0.0266
AK041838	Il7r	interleukin 7 receptor	0.319	0.0256
NM_001033126	Cd27	CD antigen 27 (Cd27), transcript variant 1	0.326	0.0158
NM_009142	Cx3cl1	chemokine (C-X3-C motif) ligand 1	0.343	0.0227
NM_010215	Il4i1	interleukin 4 induced 1	0.387	0.0129
NM_207105	H2-Ab1	histocompatibility 2, class II antigen A, beta 1	0.520	0.0255
NM_033217	Ngfr	nerve growth factor receptor (TNFR superfamily, member 16)	0.440	0.0110
AK041345	Xlr4a	X-linked lymphocyte-regulated 4A	0.460	0.0074
NM_033042	Tnfrsf25	tumor necrosis factor receptor superfamily, member 25	0.465	0.0366
NM_198297	Trat1	T cell receptor associated transmembrane adaptor 1	0.476	0.0334
NM_011161	Mapk11	mitogen-activated protein kinase 11	0.478	0.0128
NM_007549	Blk	B lymphoid kinase	0.483	0.0103
NM_010818	Cd200	Cd200 antigen	0.494	0.0156

### Genes with differential expression in brains of JEV-infected mice

Compared to the expression after mock infection, 551 genes were detected to be significantly upregulated and 568 genes were downregulated in brains of JEV-infected mice (Table 4). Consistent to the results of spleen, chemokines CCL2, CCL3, CCL4 and CXCL10, IFN response transcription factor IRF7, IFN-induced proteins like Ifit1, Ifit2 and Ifit3, protein degradation gene Usp18 were obviously upregulated, and CD163 and IRF6 showed decreased expression upon JEV infection. In addition, increased expression of IFN-inducible transcription factors STAT1 and STAT2, TNF-α induced protein TNFAIP3, apoptosis-related proteins caspase3 and caspase4, suppressors of cytokine signaling Socs1 and Socs3, pro-inflammatory cytokines IL-1 and IL-6, TLR7, as well as IFN response antiviral genes of OAS family were also observed in microarray analysis. These results suggested the occurrence of a strong inflammatory response in mouse brain.

**Table 4:** Differentially-regulated genes of our interest in brains of mice with JEV Infection.

Genbank Accession	Gene symbol	Gene description	Fold change	*p*-Value
**Up-regulated**				
NM_021274	Cxcl10	chemokine (C-X-C motif) ligand 10	1760.024	0.0020
NM_011333	Ccl2	chemokine (C-C motif) ligand 2	1387.794	0.0320
NM_013652	Ccl4	chemokine (C-C motif) ligand 4	336.804	0.0151
NM_010846	Mx1	myxovirus (influenza virus) resistance 1	229.188	0.0136
NM_145209	Oasl1	2'-5' oligoadenylate synthetase-like 1	209.863	0.0242
NM_011940	Ifi202b	interferon activated gene 202B	201.477	0.0317
NM_008329	Ifi204	interferon activated gene 204	165.395	0.0404
NM_021792	Iigp1	interferon inducible GTPase 1	159.817	0.0168
NM_011337	Ccl3	chemokine (C-C motif) ligand 3	111.943	0.0429
NM_008176	Cxcl1	chemokine (C-X-C motif) ligand 1	107.659	0.0158
AK085407	Ifi44	interferon gamma inducible protein 44	91.009	0.0062
NM_010555	Il1r2	interleukin 1 receptor, type II	89.273	0.0297
NM_021893	Cd274	CD274 antigen	87.250	0.0337
NM_008330	Ifi47	interferon gamma inducible protein 47	83.552	0.0286
NM_172648	Ifi205	interferon activated gene 205	82.479	0.0416
NM_194336	Mpa2l	macrophage activation 2 like	77.279	0.0464
NM_007609	Casp4	caspase 4, apoptosis-related cysteine peptidase	77.077	0.0451
NM_017466	Ccrl2	chemokine (C-C motif) receptor-like 2	74.783	0.0071
NM_008332	Ifit2	interferon-induced protein with tetratricopeptide repeats 2	65.247	0.0222
NM_011331	Ccl12	chemokine (C-C motif) ligand 12	65.192	0.0079
NM_033601	Bcl3	B-cell leukemia/lymphoma 3	53.003	0.0138
NM_029803	Ifi27	interferon, alpha-inducible protein 27	47.264	0.0403
NM_031168	Il6	interleukin 6	44.884	0.0106
NM_016850	Irf7	interferon regulatory factor 7	42.752	0.0463
NM_145211	Oas1a	2'-5' oligoadenylate synthetase 1A	41.831	0.0497
NM_011909	Usp18	ubiquitin specific peptidase 18	40.804	0.0052
NM_010501	Ifit3	interferon-induced protein with tetratricopeptide repeats 3	39.282	0.0008
NM_007707	Socs3	suppressor of cytokine signaling 3	36.912	0.0087
NM_008331	Ifit1	interferon-induced protein with tetratricopeptide repeats 1	32.115	0.0013
NM_013606	Mx2	myxovirus (influenza virus) resistance 2	31.018	0.0003
NM_011854	Oasl2	2'-5' oligoadenylate synthetase-like 2	29.655	0.0131
NM_009896	Socs1	suppressor of cytokine signaling 1	28.609	0.0492
NM_009140	Cxcl2	chemokine (C-X-C motif) ligand 2	24.678	0.0392
NM_010397	H2-T22	histocompatibility 2, T region locus 22	21.010	0.0473
NM_027835	Ifih1	interferon induced with helicase C domain 1	19.804	0.0233
NM_009397	Tnfaip3	tumor necrosis factor, alpha-induced protein 3	19.385	0.0300
NM_009283	Stat1	signal transducer and activator of transcription 1	18.134	0.0348
NM_001083925	Oas1b	2'-5' oligoadenylate synthetase 1B	17.574	0.0342
NM_008361	Il1b	interleukin 1 beta	15.753	0.0043
NM_133211	Tlr7	toll-like receptor 7	9.880	0.0343
NM_027450	Glipr2	GLI pathogenesis-related 2	9.275	0.0405
NM_008562	Mcl1	myeloid cell leukemia sequence 1	9.054	0.0336
NM_029419	Apol3	apolipoprotein L 3	8.806	0.0165
NM_001008700	Il4ra	interleukin 4 receptor, alpha	8.639	0.0360
NM_013730	Slamf1	signaling lymphocytic activation molecule family member 1	8.274	0.0435
NM_009841	Cd14	CD14 antigen	6.666	0.0034
NM_033541	Oas1c	2'-5' oligoadenylate synthetase 1C	6.596	0.0120
NM_009810	Casp3	caspase 3, apoptosis-related cysteine peptidase	2.119	0.0117
**Down-regulated**				
NM_053094	Cd163	CD163 antigen	0.066	0.0007
XM_898059	Cd209f	CD209f antigen	0.088	0.0271
NM_026972	Cd209b	CD209b antigen	0.132	0.0149
NM_033042	Tnfrsf25	tumor necrosis factor receptor superfamily, member 25	0.196	0.0288
NM_016708	Npy5r	neuropeptide Y receptor Y5 (Npy5r)	0.242	0.0351
NM_019577	Ccl24	chemokine (C-C motif) ligand 24 (Ccl24)	0.302	0.0132
NM_008409	Itm2a	integral membrane protein 2A	0.302	0.0075
AK042749	D230046B21Rik	7 days neonate cerebellum cDNA, RIKEN full-length enriched library, clone:A730020N04	0.311	0.0358
NM_030143	Ddit4l	DNA-damage-inducible transcript 4-like	0.318	0.0004
NM_022723	Scube1	signal peptide, CUB domain, EGF-like 1	0.319	0.0400
AK144387	4732444A12Rik	21 days neonate cerebellum cDNA, RIKEN full-length enriched library, clone:G630051C23	0.319	0.0000
AK082652	Tmem44	transmembrane protein 44	0.324	0.0108
NM_175106	Tmem177	transmembrane protein 177 (Tmem177)	0.330	0.0346
NM_175564	Tmem169	transmembrane protein 169	0.336	0.0371
NM_027016	Tloc1	translocation protein 1	0.336	0.0276
NM_027163	Il1f8	interleukin 1 family, member 8	0.421	0.0267
NM_022986	Irak1bp1	interleukin-1 receptor-associated kinase 1 binding protein 1	0.422	0.0169
NM_016851	Irf6	interferon regulatory factor 6	0.447	0.0303
NM_145826	Il17re	interleukin 17 receptor E (Il17re), transcript variant 1	0.477	0.0403

### Confirmation of microarray data by RT-qPCR

To confirm the microarray hybridization results, quantitative RT-PCR was performed on eight selected differentially expressed mRNAs in mouse spleen and brain respectively. As shown in the RT-qPCR result of spleen, granzymA, granzymeB, Porferin, IRF7, IFN-γ, CXCL10, and ILIR2 were significantly upregulated, while CD163 was downregulated (Table 5). Out of eight tested mRNAs in brains of mice infected with JEV, CCL2, CCL4, CXCL10, Casp3, Casp4, SOCS1 and SOCS3 showed increased expression, and CD163 was also found to have an obviously decreased expression (Table 5). Although absolute values are not identical due to the different sensitivity between the techniques, all genes showed a well comparative expression pattern with microarray data.

**Table 5:** Comparison of expression changes of some selected genes between microarray and qRT-PCR

Gene name	Gene description	Fold change	
		Microarray	qRT-PCR
**Spleen**			
Cxcl10	chemokine (C-X-C motif) ligand 10	3.418	3.375 (±1.065)
Ifng	interferon gamma	2.229	3.062 (±0.507)
Gzmb	granzyme B	6.202	7.285 (±0.311)
Gzma	granzyme A	4.236	3.815 (±0.127)
Prf1	Porferin 1	1.820	2.287 (±0.643)
Irf7	interferon regulatory factor 7	3.378	4.926 (±0.309)
Il1r2	Interleukin 1 receptor, type II	2.069	2.078 (±0.105)
Cd163	CD163 antigen	0.060	0.083 (±0.017)
**Brain**			
Cxcl10	chemokine (C-X-C motif) ligand 10	1760.024	13.492 (±1.690)
Ccl2	chemokine (C-C motif) ligand 2	1387.794	188.549 (±8.931)
Ccl4	chemokine (C-C motif) ligand 4	336.804	61.007 (±3.735)
Casp3	Caspase3, apoptosis-related cysteine peptidase	2.119	1.207 (±0.073)
Casp4	caspase4, apoptosis-related cysteine peptidase	77.077	19.969 (±0.050)
Socs1	suppressor of cytokine signaling 1	28.609	11.182 (±0.845)
Socs3	suppressor of cytokine signaling 3	36.912	4.672 (±0.464)
Cd163	CD163 antigen	0.066	0.065 (±0.012)

## Discussion

Spleen is one of the major peripheral immunity organs and CNS is the ultimate infection target of JEV. Therefore, identification of the JEV-related host genes in spleens and brains of mice infected with JEV may be helpful for understanding of JEV pathogenesis. To this end, profiles of mRNA expression in both spleen and brain tissues of JEV-infected mice were analyzed systematically in this study.

In mRNA profiling assay, we found chemokines like CCL2 and CXCL10 were significantly up-regulated in both mouse spleen and brain in response to JEV-infection, suggesting a strong inflammatory response of host. Monocyte chemoattractant protein-1 (MCP-1/CCL2) is one of the key chemokines that regulate migration and infiltration of monocytes/macrophages. It was involved in neurological disorders such as encephalitis-related neuronal death, where its levels were elevated in astrocytes leading to neuronal death [13]. Previous study has demonstrated that the WNV-infection stimulated the expression of CCL2 in mice livers, suggesting a consistent result to our study of JEV [10]. CXCL10 is also found to play a crucial role in the host defense response against various viral infection of the CNS by enhancing innate immune responses [14,15], and our result of up-regulated CXCL10 mRNA has a good agreement with that was reported by Gupta et al. and Biswas et al. [11,12].

In addition, a strong IFN-pathway-related response was evident in mouse spleen and brain infected with JEV, with increased expression of IFN-γ, IFN response transcription factor IRF7, IFN-induced proteins IFIT1,IFITM3 and IFITM7 in spleen, and IRF7, IFN-inducible transcription factors STAT1 and STAT2, IFN-induced proteins IFIT1, IFIT2 and IFIT3 in brain, implying the occurrence of a protective response of host. Similar results were shown in previous reports as the upregulation of IFN-γ, STAT1 and STAT2 in JEV-infected mice brain [12], and increased expression of STAT1 and IFIT3 in JEV-infected N2A cells [11]. The reason why no increase in expression of IFN-γ was found in brains of JEV-infected mice in our study may be related to the difference of time points with Biswas' study [11]. IRF7 is activated in the presence of double stranded RNA following virus infection, which is functional as one of the regulators of the IFN-α/β gene promoter and the IFN-α/β responsive genes to create an antiviral state [16]. The increased expression of IRF7 has also been demonstrated in WNV infected mice, but wasn't found in JEV infected cells [17,18]. This may be due to the different signal pathways between intact host and cell culture.

Pore-forming protein perforin and the family of granzymes have been demonstrated to form an antiviral arsenal central to the function of cytotoxic T lymphocyte (CTL) and natural killer (NK) cells [19-21]. After JEV infection, pro-apoptotic genes found to have significant enhancement in mouse spleen, including porferin, granzyme A and granzymeB, which suggested a strong cytotoxic response against JEV infection. The consistent result as increased expression of granzyme A and B was also shown in the report about WNV infection [17]. Granzyme B is the most characterized granyzme which plays an important role in inducing apoptosis, and it is generally accepted that granzyme A can trigger a distinct nonapoptotic form of cell death [22]. The high expression of granzyme A and B in spleen could help clear virus infection, but may also involve lymphocyte injury. The upregulation of apoptosis-related proteins Caspase3 and 4 were also detected in brains of mice infected with JEV, indicating an inflammation-related neuronal apoptosis. Caspase 3 is an effector caspase that function as a central regulator of apoptosis. It has been reported that JEV infection triggers apoptosis in different cells, such as baby hamster kidney BHK-21 cells, mouse neuroblastoma N18 cells, human neuronal NT-2 cells, and human medulloblastoma cells, resulting in caspase 3 activation [23-25]. The function of caspase 4 is not fully known, but it is believed to be an inflammatory caspase, with a role in the immune system [26].

Pro-inflammatory cytokines like IL-1 and IL-6 were found to have increased expression in the brain, which was consistent with the results of the studies on DV and WNV [8,17]. IL-1α and IL-1β has been known to form an important part of the inflammatory response of the body against infection. IL-6 together with IL-1 and TNF-α acts as an endogenous pyrogen by causing fever following viral infections [27]. It's also associated with an unfavorable outcome following yellow fever virus infection [28]. Evidence suggests that circulating IL-6 can activate CNS mechanisms resulting in the development of the febrile response during disease [29]. Upregulation of IL-1 and IL-6 in brain may thus be protective against harmful JEV infection, but also have a pathogenic role in CNS.

The 2', 5'-oligoadenylate synthetase (OAS) and its downstream effector RNase L play important roles in host defense against virus infection. The OAS1b protein has been described as a flavivirus resistance factor, and OASl1 as a WNV-resistance factor in wild mice because a truncated version of the protein is expressed in laboratory mice which are susceptible to infection [30-32]. Human OAS1 p42/p46 and OAS3 p100 are likely to contribute to host defense against DEN infection and play a role in determining the outcomes of DEN disease severity [33]. Further, the activated expression of OAS2 has been demonstrated in mouse brain in response to JEV infection [34]. In present study, there was increased expression of various members of the OAS family in the brains of JEV-infected mice, including OAS1a, OAS1b, OAS1c, OAS1e, and OAS2. Therefore, the ability of these proteins to protect against JEV infection should also be further studied.

The significant downregulation of CD163 was detected both in mouse spleen and brain in response to JEV infection. CD163 is a novo identified marker for perivascular macrophages in humans, monkeys, and mice. And previously studies have found that perivascular CD163 expression is upregulated and the number of CD163-positive cells increases in HIV and SIV encephalitis (HIVE and SIVE) brains [35,36]. However, CD163 is not a "classical" activation marker, because peripheral blood monocytes and most tissues macrophages of normal uninfected controls all express it and because *in vitro *pro-inflammatory stimuli largely down-regulate its expression. These findings suggest that CD163 expression is regulated in association with a certain stage of differentiation. Here, our results showed a downregulated CD163 mRNA level in response to acute JEV infection, which probably suggested a decreased number of activated perivascular macrophages resulted by inflammatory disorder-related apoptosis response.

## Conclusions

In summary, our findings suggested that JEV infection resulted in significant changes in the expression of multiple genes in mouse spleen and brain, including inflammatory cytokines, chemokines, IFN inducible genes, IFN regulators, and apoptosis related genes, etc. These genes may play a critical role on antiviral response of host against JEV infection but could also contribute to the pathogenesis of JEV resulting in encephalitis. The mRNA profile obtained by microarray analysis in this study may provide a foundation for future investigation of JEV pathogenesis and therapeutic method.

## Methods

### Virus production

JEV wide type strain P3 was propagated in brains of suckling mice and titered in BHK-21 cells which was grown and maintained in Dulbecco's Modified Eagle's Medium (DMEM) supplemented with 10% heated-inactivated fetal bovine serum (FBS, Hyclone, Logan, UT, USA), 100 g/ml streptomycin and 100 IU/ml penicillin (Sigma-Aldrich, MO, USA) at 37°C with 5% CO2.

### Virus infection of mice

Four-weeks-old naive female BALB/c mice were purchased from Wuhan Institute of Biologic Products (Hubei Province, China) and inoculated subcutaneously (s.c.) with 5 × 10^6 ^PFU of JEV P3 strain. Dilutions were performed in serum-free DMED and experimental controls were mock injected with diluent. A part of JEV-infected mice were sacrificed at day 3 post-inoculation (the day before the neurological symptoms appeared), and spleens were harvested. The left mice were sacrificed at day 6 post-inoculation (the day before the mice started to die), and brains of mice were harvested. Spleen and brain homogenate was made in DMEM for RNA extraction.

### Microarrays and bioinformatics

The total RNA was isolated from mouse spleens and brains respectively with trizol reagent (Invitrogen) for mRNA Microarray. mRNA hybridization was performed by shanghaiBio Corporation (shanghai, China) with the use of 4 × 44 K Agilent Whole Mouse Genome Oligo Microarray (total 41,174 oligo probes from 41,174 mouse genes). For each sample pair, the experiments were done with two independent hybridizations (Cy3 and Cy5 interchanging labeling). Hybridized arrays were scanned at 5 μm resolution on an Agilent DNA Microarray Scanner (Model G2565BA). Data extraction from images was done by using Agilent Feature Extraction software. Hierarchical cluster, gene ontology and pathway analysis were analyzed by using SAS (ShanghaiBio Analysis System).

### Quantitative real-time RT-PCR (RT-qPCR)

For selected mRNA RT-qPCR, total RNA from the same samples used in microarray analysis was tested by using ABI 7500 FAST real-time PCR system. PCR primers were designed with Primer Express 2.0 software (Invitrogen). Results are shown as fold change. For mRNA RT-qPCR, experiments were carried out with the PrimerScript RT reagent Kit (TaKaRa) and SYBR Green Realtim PCR Master Mix (TaKaRa) according to manufacture's instruction. The housekeeping gene GHDAP was used for standardization of the initial RNA content of a sample. Relative changes of gene expression were calculated by the following formula, and the data are represented as fold upregulation/downregulation. fold change = 2^-ΔΔCt^, whereΔΔCt =(Ct of gene of interest, treated -Ct of HK gene, treated)-(Ct of gene of interest, control-Ct of HK gene, control), Ct is the threshold cycle number and HK is the house keeping gene.

### Statistical analysis

Each gene in each infection group was subjected to a Student's t test to detect large expression differences relative to the mock-infected group. *p*-values <0.05 were considered to be statistically significant.

## Competing interests

The authors declare that they have no competing interests.

## Authors' contributions

YY and JY carried out most of the experiments and wrote the manuscript. XY, RJ participated part of experiments. HC and SC conceived of the study, participated in its design and coordination, and revised the manuscript. All authors read and approved the final manuscript.
